# Expected climate change consequences and their role in explaining individual risk judgments

**DOI:** 10.1371/journal.pone.0281258

**Published:** 2023-02-15

**Authors:** Thea Gregersen, Rouven Doran, Gisela Böhm, Hans-Rüdiger Pfister

**Affiliations:** 1 Department of Psychosocial Science, University of Bergen, Bergen, Norway; 2 Centre for Climate and Energy Transformation (CET), University of Bergen, Bergen, Norway; 3 Norwegian Research Centre (NORCE), Bergen, Norway; 4 Department of Psychology, Inland Norway University of Applied Sciences, Lillehammer, Norway; 5 Institute of Experimental Industrial Psychology (LueneLab), Leuphana University Lüneburg, Lüneburg, Germany; Texas A&M University, UNITED STATES

## Abstract

This study examines what individuals expect will be the most important impacts of climate change on their respective countries, and how these expectations relate to individual risk judgments. Open-ended responses from representative samples in four European countries (each *n* > 1000), were sorted into six categories: expectations of climate change leading to changes in attitudes and goals, human activities, emissions and pollution, environmental changes, impacts on humans, or few or no impacts. The results showed that the most frequently mentioned climate change impacts were related to environmental changes. Although most results were consistent across the UK, Norway, Germany, and France, some differences were identified. For example, respondents in the UK and Norway more frequently mentioned changes in human actions and activities among the most important climate change impacts. We also found differences between demographic groups; men, those in the oldest age groups, and those placing themselves further right on the political spectrum were more likely to expect few or no consequences of climate change on their country. Additional analyses examined relationships between the six impact categories and two different measures of individual risk judgments. Those expecting climate change to lead to changes in attitudes and goals, environmental changes, or impacts on humans reported higher levels of worry about climate change and expected more negative effects on their country. Climate change worry, but not the evaluation of how positive or negative effects will be on one’s country, was further related to the number of consequences mentioned in response to the open-ended question and the specificity conveyed.

## Introduction

Direct and indirect consequences of climate change range from biodiversity loss, flooding and heat waves, to changes in energy production, infrastructure and agriculture [1]. The extent to which people perceive climate change as a risk is associated with their beliefs about its potentially negative impacts [e.g., 2–4]. One obstacle to public engagement is that people tend to perceive climate change as a threat that will mostly affect spatially distant places [5–7]. Because of this, people’s assessments of how negative the effects of climate change will be on their respective countries are likely to differ from their general worry about climate change. The present study sets out to explore unprompted responses when people are asked about their expectations regarding the consequences of climate change for their own country, in addition to exploring if an emphasis on specific consequences contributes to explaining differences in individual risk judgments. While several studies have investigated the public’s understanding of the climate system, these have mainly focused on identifying prevailing misconceptions rather than studying how subjective conceptions about climate-related events may shape risk judgments [e.g., 8–10].

Böhm and Pfister [11] propose that people’s mental representation of environmental risks can be described with a multi-level framework focusing on the risks’ causal structure. The framework includes five consecutive ‘levels’, spanning from attitudes and goals to human activities, emissions and pollution, environmental changes, and finally impacts on humans. A societal-level example of this structure is how a desire for economic growth can lead to continued oil and gas production, which leads to carbon dioxide emissions, causing increased climate change-induced flooding incidents, and resulting in damage to buildings. The five levels are assumed to be circular; that is, people’s motives and goals form the basis for certain actions (e.g., driving by car, adopting certain technologies); the consequences of these actions harm the environment, which leads to impacts on humans. Experiencing these impacts might create new motives and goals, for example towards adaptive or mitigative actions. Consequently, changes in attitudes and goals can be conceptualized as both a cause for human activities and a consequence of experienced impacts. The depicted framework is based on Hohenemser, Kasperson and Kates’ [12] conceptualization of the causal structure of hazard control, which understands environmental risks as a causal chain of events and assumes that the chain can be blocked by exerting control in the transitions between levels.

The subjective conception of climate-related events, and specifically simplified cognitive representations of causes and consequences, are central for climate change risk perception [13]. Hence, we anticipate that expected climate change consequences will relate to people’s general worry about climate change and their evaluation of how negative climate change will be for their own country. Previous research has found that people tend to mention consequences related to emissions and pollution (e.g., air pollution), and environmental changes (e.g., ozone depletion) when asked about what they consider to be global environmental *risks* [14]. Expectations of possible negative consequences for humans are believed to be a core characteristic of why people identify a given phenomenon as a risk and report higher levels of emotions such as worry [11]. However, human *activities* might be perceived as somewhat controllable and therefore seen as less of a risk [15]. We take an explorative approach to investigate the relationship between what people believe will be the most important impact of climate change on their country and their risk judgments. In addition to possible differences in the *content* laypeople emphasize, causal beliefs can also differ concerning their underlying *structure* [see e.g., 16–18]. In line with this, we will look at how mentioning impacts across (or within) each level is related to individual risk judgments.

Although the consequences of climate change will be mostly negative across the world, vulnerability towards specific impacts can differ both between countries and between groups within a country [1]. Further, risk perception is to some extent socially constructed, depending on social, cultural, economic, and political influences [19]. Given that risks are (partly) socially constructed, the same risk can be evaluated differently at different times, in different places, and by different groups. Support for this is provided by research demonstrating cross-national differences when people are asked what they associate with ‘climate change’ in general [20], and that country-level conditions (e.g., economic prosperity, democracy, exposure to climate impacts) tend to be related to climate change beliefs [21, 22]. Studies further suggest that the political and institutional responses to climate change events, as well as corresponding media coverage, can influence the public’s perception of risk [23, 24]. People living in countries less ‘vulnerable’ to direct climate change impacts may have other impact associations and focus on a broader range of consequences, including indirect effects such as changes in attitudes or activities. For example, the rapid shift to electric cars [25] or the potential for reduced oil production might be perceived as important consequences of climate change in Norway. We assume that expected climate change consequences may differ across countries and sociodemographic groups.

The current paper includes impact expectations from people in four key European countries: the UK, Norway, Germany, and France [26]. The overarching aim of the present investigation was threefold: (i) to describe expectations about possible consequences of climate change according to Böhm and Pfister’s framework across the four countries, (ii) to investigate whether impact expectations can be predicted from socio-demographic features such as age, gender, and political orientation, and finally (iii) to explore the relationship between impact expectations and climate change worry in general and/or evaluation of how negative climate change will be for one’s country. The range of possible impact expectations might be especially relevant if studying countries that vary in per capita emissions and energy production as well as in the degree to which they have been and potentially will be impacted by climate change events; for an overview of the socio-political profile of each included country, see [27].

## Materials and method

### Data collection

The data were collected as part of a research project on public perceptions of climate change [28]. The countries included in the project (UK, Norway, Germany, and France) are among the most important energy-producing countries in Europe, they have large and varied energy systems and decisions made in these countries could strongly influence Europe’s energy transition [26]. The design of the study was informed by an analysis of the socio-political contexts within each of the included countries. The profiles include information about culture, history, policy, influential actors, media coverage, and past and anticipated climate change events [27]. The survey was developed in English and later translated into the other three national languages. Representative samples of about 1000 respondents aged 15 and over from each of the participating countries were collected via face-to-face interviews (UK, Germany, and France) or telephone interviews (Norway). The data collection was administered by Ipsos Mori, an international survey company. The company followed a sampling scheme in each of the countries that ascertained representative samples, with sampling points distributed across the country and quotas set to demographic variables such as age, gender, education, and region. A detailed description of the methodology and sampling procedure can be found in [26, 28]. The survey included both open- and closed-ended survey questions, and took between 22 and 28 minutes to complete on average. Fieldwork was completed in June 2016. More detailed information on sample characteristics, project design, methodology, and other questions included in the survey can be found elsewhere [26].

All procedures were in accordance with the ethical standards of the School of Psychology Ethics Committee (Cardiff University) and with the 1964 Helsinki Declaration and its later amendments.

### Measurements

The survey included an open-ended question so that respondents could answer in an unprompted and unrestricted manner based on their immediate and subjective associations, understandings, and feelings about the effects of climate change. The exact formulation was: ‘Climate change may affect different countries in different ways. What do you think will be the most important effect of climate change on [France/Germany/Norway/the UK]?’.

Personal worry about climate change was assessed by asking respondents ‘How worried, if at all, are you about climate change?’, which could be answered on the following scale: 1 (Not at all worried), 2 (Not very worried), 3 (Fairly worried), 4 (Very worried), 5 (Extremely worried). There were *n =* 28 missing responses to this question, including those choosing the answer options ’Don’t know’ or ‘Refuse to respond’; the latter option was included only in France. The question ‘Overall, how positive or negative do you think the effects of climate change will be on [France/Germany/Norway/the UK]?’ was used as a risk evaluation measure using the following scale: 1 (Entirely positive), 2 (More positive than negative), 3 (Neither positive nor negative), 4 (More negative than positive) and 5 (Entirely negative). ‘Don’t know’ and ‘Refuse to respond’ were categorized as missing (*n =* 198). Respondents who answered ‘there is no such thing as climate change’ on an earlier question in the survey were not asked to answer the open-ended question or to indicate how positive or negative the impacts of climate change will be on their country.

Socio-demographic characteristics were included in the analyses based on literature identifying gender, age, and political orientation as relevant for explaining individual differences in climate change beliefs [see e.g., 29]. Gender (0 = Men, 1 = Women) and age (0 = 15–24, 1 = 25–34, 2 = 35–44, 3 = 45–54, 4 = 55–64, 5 = 65+) were treated as categorical variables. Political orientation was measured with the question: ‘In politics people sometimes talk of “left” and “right”. Using a scale from 0 to 10, where 0 means the left and 10 means the right, where would you place yourself on this scale?’ (*n =* 399 missing values, including the answers ’Don’t know’ and ‘Refuse to respond’).

A summary of descriptive statistics and correlations among closed-ended questions can be found in the supporting information, S1 Table.

### Statistical analysis

A coding scheme was developed in German and later translated into Norwegian, English, and French. Using content analysis [30], responses were sorted into six categories, five of which correspond to the causal levels described in Böhm and Pfister’s [11] multi-level framework. The sixth category included answers reflecting expectations of hardly any impacts of climate change on one’s own country; for more details, see Table 1. Subcategories were used in some cases to characterize the responses more specifically or to differentiate between relevant characteristics. For example, a response categorized as Level 4 could either reflect a rather abstract answer such as ‘We hardly have seasons anymore’, or a more concrete answer specifying that the consequence would influence plants ‘Crops like corn will benefit, others will yield less harvest. Pests attacking plants will multiply’ (subcategory 1). Each response could be sorted into one, several, or none of the categories, depending on how many categories were expressed by the respondent. An overview of the coding scheme, including all subcategories, is provided in the supporting information, S2 Table.

**Table 1:** Frequencies for causal beliefs about climate change within the four countries (weighted).

Categories	Example responses	UK	Norway	Germany	France	Total
Attitudes, motives, and goals (Level 1)	«People will finally wake up»	14.95 %	0.49 %	1.28 %	1.24 %	4.68%
Actions and activities (Level 2)	«We will save more energy at home»	14.80 %	8.66 %	4.72 %	3.66 %	8.05%
Emissions and pollution (Level 3)	«CO_2_-emissions and CFCs»	12.92 %	6.17 %	2.50 %	7.92 %	7.53%
Environmental changes (Level 4)	«We hardly have seasons any more»	60.94 %	85.93 %	86.81 %	85.92 %	79.55%
Impacts on humans (Level 5)	«There will be severe consequences for us humans»	9.85 %	12.84 %	19.15 %	15.28 %	14.18%
Hardly any impacts	«Nothing will change»	11.86 %	2.93 %	1.63 %	0.28 %	4.29%

Note. Each answer to the open-ended question could be sorted into several levels. Example responses are retrieved from the coding scheme available in the S2 Table. Missing responses were removed before calculating the percentages.

The coding was conducted independently by two native speakers of the country’s language. Intercoder reliability was assessed as proportion agreement, calculated as the number of codings where the two coders gave the same code divided by the total number of codings. The interrater reliability was 0.924 for the UK, 0.972 for Norway, 0.982 for Germany, and 0.976 for France. The two coders in each country were later instructed to discuss whether they could resolve any of the coding disagreements. After solving disagreements to the extent possible, interrater reliability increased to 1.0 in the UK and 0. 999 in Norway, Germany, and France. The complete coding instructions are included in the supporting information, S1 File. Example responses can be seen in Table 1.

Logistic regressions were used to investigate whether gender, age, and political orientation predicted the likelihood of mentioning specific categories while controlling for the country variable. We fit a total of six regression models, one for each category. In the next step, we used multiple linear regressions to explore the relationship between the content of the obtained responses (i.e., the six categories described above) and individual risk judgments (i.e., worry and risk evaluation), while controlling for the effect of several covariates (i.e., gender, age, political orientation, and country). The six categories used to code the open-ended responses were included as dichotomous variables (0 = category does not apply, 1 = category does apply).

Additional multiple linear regressions tested possible effects concerning the structure of the obtained responses. An index for width was created by summarizing how many of the five causal levels from Böhm and Pfister’s [11] framework were mentioned in each response (i.e., Level 1 + Level 2 + Level 3 + Level 4 + Level 5; see S2 Table for the numbering of the levels), while excluding answers that mentioned either hardly any impacts or that did not relate to any of the categories (*n* = 134). Indexes for depth were calculated by looking at the level of specificity at the first sub-level (i.e., Level21 + Level22 + Level41 + Level42 + Level51 + Level52) and at the second sub-level (i.e., Level211 + Level212 + Level213 + Level221 + Level222 + Level223 + Level511 + Level521 + Level522 + Level523). Gender, age, political orientation, and country were included as covariates. The dataset was merged across countries for all regression analyses.

All analyses were conducted in STATA 16.1 using the survey prefix command svy: to include survey weights in the regressions. Weights were added to ensure that the samples are representative of the adult population in each of the four countries. Using the svy: prefix gives coefficient *p*-values based on the Wald test. Effect sizes (McFadden’s Pseudo R^2^) for the logistic regressions were calculated from non-weighted versions of the regression models.

## Results

### Country differences in expected climate change consequences

Most respondents (79% in the UK, 84% in Norway, 84% in Germany, and 88% in France) whose answers could be sorted into the six categories gave a response that reflected only one category. Table 1 shows that the most frequently mentioned climate change consequence concerned environmental changes (Level 4), comprising about 61% of the responses in the UK, 86% in Norway and France, and 87% in Germany. There are some country differences concerning what kind of environmental changes people mention (see S3 Table). Respondents in Germany (38%) and France (41%) more frequently specified natural disasters as the most important impact than did respondents in the UK (9%) and Norway (13%). Especially in Norway, the mentioned ‘environmental changes’ were often more general, for example by mentioning temperature or weather changes (while 85% of the respondents mention environmental changes, only 23% specify natural disasters or impacts on animals and plants).

While impacts on humans (Level 5) were the second most frequently mentioned category across countries (14% in total), and in both Norway, Germany, and France, the second most frequently mentioned category in the UK was attitudes, motives, and goals (Level 1). The least frequently mentioned category was impacts on humans (Level 5) in the UK, attitudes, motives, and goals (Level 1) in Norway and Germany, and hardly any impacts in France. The number of respondents mentioning hardly any impacts varied considerably across countries, comprising about 12% of responses in the UK and less than 1% in France. Frequencies for all sub-levels can be seen in the supporting information, S3 Table.

### Socio-demographic differences in expected climate change consequences

Table 2 shows that women were significantly more likely than men to mention environmental changes (Level 4) and significantly less likely to expect hardly any impacts. Further, those who placed themselves further right on the political spectrum were less likely to mention impacts on humans (Level 5) and more likely to expect hardly any impacts. Those aged 65+, as compared to those aged 15–24, were less likely to mention environmental changes. Respondents aged 65+ or aged 55–64 were more likely to answer that climate change will have hardly any impact on their country compared to participants aged 15–24. The socio-demographic variables had no significant effect on mentioning any of the other levels. Country was included as a control variable, and its effects in the regression complement those reported in the frequency table above (see Table 1). The socio-demographic- and country variables explained far more of the variance in category 1 and 6 as compared to the others, although only country had a significant effect on mentioning attitudes, motives and goals.

**Table 2:** Predicting the content of causal beliefs about climate change from gender, age, political orientation, and country (weighted).

	Attitudes, motives, and goals (Level 1)	Actions and activities (Level 2)	Emissions and pollution (Level 3)	Environmental changes (Level 4)	Impacts on humans (Level 5)	Hardly any impacts
	*B (SE)*	*B (SE)*	*B (SE)*	*B (SE)*	*B (SE)*	*B (SE)*
Intercept	-1.35 (0.30)***	-1.69 (0.25)***	-2.19 (0.27)***	0.66 (0.18)***	-2.03 (0.23)***	-3.36 (0.46)***
Gender						
Women	-0.16 (0.17)	0.07 (0.13)	0.08 (0.14)	0.20 (0.09)*	0.07 (0.11)	-0.61 (0.19)**
Age						
25–34	-0.30 (0.35)	-0.33 (0.27)	0.45 (0.27)	-0.28 (0.19)	0.22 (0.23)	0.75 (0.45)
35–44	-0.24 (0.35)	-0.10 (0.25)	-0.01 (0.28)	-0.14 (0.19)	0.41 (0.21)	0.18 (0.49)
45–54	0.20 (0.31)	-0.07 (0.24)	0.14 (0.26)	-0.24 (0.18)	0.27 (0.22)	0.60 (0.44)
55–64	-0.25 (0.32)	-0.04 (0.25)	0.24 (0.26)	-0.34 (0.18)	0.06 (0.22)	0.89 (0.44)*
65 +	0.33 (0.28)	-0.15 (0.23)	0.01 (0.25)	-0.35 (0.17)*	0.39 (0.21)	1.35 (0.41)**
Political orientation	-0.06 (0.04)	0.01 (0.03)	0.02 (0.03)	-0.02 (0.02)	-0.09 (0.02)***	0.17 (0.05)**
Country						
Norway	-3.56 (0.52)***	-0.60 (0.16)***	-0.81 (0.19)***	1.40 (0.13)***	0.30 (0.16)	-1.60 (0.25)***
Germany	-2.53 (0.35)***	-1.23 (0.21)***	-1.81 (0.25)***	1.43 (0.13)***	0.76 (0.16)***	-2.11 (0.33)***
France	-2.51 (0.32)***	-1.50 (0.21)***	-0.57 (0.18) **	1.40 (0.13) ***	0.47 (0.16) **	-4.44 (1.01)***
Variance explained	Pseudo *R*^*2*^ = .21	Pseudo *R*^*2*^ = .05	Pseudo *R*^*2*^ = .04	Pseudo *R*^*2*^ = .08	Pseudo *R*^*2*^ = .02	Pseudo *R*^*2*^ = .20

Note. Political orientation is continuous, gender is dichotomous (men as reference category), and age (age group 15–24 as reference category) and country (UK as reference category) categorical. All reported p-values are based on the Wald test.

*p < .05

**p < .01

***p < .001

### Predicting individual risk judgments from expected climate change consequences

A closer look at the data indicated that respondents in the four countries differed regarding their level of worry about climate change. While about 42% of the respondents in France reported being ‘very worried’ or ‘extremely worried’ about climate change, the same was true for 30% in Germany, 29% in Norway, and 19% in the UK. There were also differences in expectations of how positive or negative the effects of climate change will be on one’s own country. The percentage of respondents reporting that they believe the effects will be ‘more negative than positive’ or ‘entirely negative’ was 75% in France, 79% in Germany, 57% in Norway, and 57% in the UK. A complete overview of survey responses can be found elsewhere [26]. Means and standard deviations of worry and risk evaluation for the full sample in each of the countries are depicted in Table 3; the two measures correlate moderately, *r*(3830) = .30, *p* < .001.

**Table 3:** Means and standard deviations for worry about climate change and risk evaluation by country (weighted).

	Worry about climate change		Risk evaluation	
	*M*	*SD*	*M*	*SD*
UK	2.73	1.10	3.58	0.94
Norway	3.01	0.96	3.55	0.91
Germany	2.96	1.09	4.02	0.81
France	3.29	1.03	3.90	0.86

The relationship between the level at which respondents identified climate change consequences for their country and individual risk judgments differed between the outcome measures (see Table 4). Higher levels of worry were positively related to mentioning consequences regarding attitudes, motives and goals (Level 1), emissions and pollution (Level 3), environmental changes (Level 4), and impacts on humans (Level 5), and negatively related to hardly any impacts. Furthermore, worry was higher among women and those who placed themselves further left on the political spectrum. Risk evaluation was positively related to attitudes, motives and goals (Level 1), environmental changes (Level 4), and impacts on humans (Level 5), as well as positively related to gender (women). It was also negatively associated with mentioning hardly any impacts, belonging to any of the three oldest age groups, and placing oneself further right on the political spectrum. These variables explained about 14% of the variance in worry about climate change and about 9% of the variance in risk evaluation.

**Table 4:** Predicting worry about climate change and risk evaluation from the content of causal beliefs about climate change (weighted).

	Worry about climate change	Risk evaluation
	*B (SE)*	*B (SE)*
Intercept	2.82 (0.09)***	3.78 (0.09)***
Attitudes, motives, and goals (Level 1)	0.52 (0.10)***	0.23 (0.09)*
Actions and activities (Level 2)	0.05 (0.07)	-0.13 (0.08)
Emissions and pollution (Level 3)	0.29 (0.07)***	0.12 (0.07)
Environmental changes (Level 4)	0.18 (0.06)**	0.18 (0.06)**
Impacts on humans (Level 5)	0.19 (0.06)**	0.13 (0.05)**
Hardly any impacts	-0.63 (0.10)***	-0.22 (0.10)*
Gender		
Women	0.27 (0.04)***	0.08 (0.03)*
Age		
25–34	0.11 (0.07)	-0.04 (0.06)
35–44	0.09 (0.07)	-0.07 (0.06)
45–54	0.00 (0.06)	-0.21 (0.06)***
55–64	0.07 (0.07)	-0.23 (0.06)***
65+	-0.04 (0.06)	-0.31 (0.06)***
Political orientation	-0.08 (0.01)***	-0.04 (0.01)***
Country		
Norway	0.31 (0.05)***	-0.02 (0.05)
Germany	0.29 (0.06)***	0.36 (0.05)***
France	0.52 (0.06)***	0.28 (0.05)***
Variance explained	*R*^*2*^ = .14	*R*^*2*^ = .09

Note. *n =* 3228 for worry about climate change and *n* = 3169 for risk evaluation. Political orientation is continuous, gender is dichotomous (men as reference category), and age (age group 15–24 as reference category) and country (UK as reference category) categorical. All reported p-values are based on the Wald test.

*p < .05

**p < .01

***p < .001

The number of mentioned categories, and the first sub-level depth index, were positively related to worry about climate change (see Table 5). None of the structural indices was related to risk evaluation. In sum, the tested regression models explained about 9% of the variance in worry about climate change and about 8% of the variance in risk evaluation, with each model controlling for gender, age, political orientation, and country.

**Table 5:** Predicting worry about climate change and risk evaluation from the structure of causal beliefs about climate change (weighted).

	Worry about climate change	Risk evaluation
	*B (SE)*	*B (SE)*
Intercept	2.94 (0.09)***	3.86 (0.08)***
Width	0.11 (0.05)*	0.07 (0.05)
Depth 1	0.10 (0.03)**	0.05 (0.03)
Depth 2	-0.02 (0.05)	-0.07 (0.05)
Gender		
Women	0.26 (0.04)***	0.10 (0.03)**
Age		
25–34	0.08 (0.07)	-0.06 (0.06)
35–44	0.06 (0.07)	-0.07 (0.06)
45–54	-0.01 (0.06)	-0.23 (0.06)***
55–64	0.05 (0.07)	-0.25 (0.06)***
65+	-0.05 (0.07)	-0.33 (0.06)***
Political orientation	-0.08 (0.01)***	-0.04 (0.01)***
Country		
Norway	0.25 (0.05)***	0.01 (0.05)
Germany	0.24 (0.06)***	0.40 (0.05)***
France	0.45 (0.05)***	0.30 (0.05)***
Variance explained	*R*^*2*^ = .09	*R*^*2*^ = .08

Note. *n =* 3092 for worry about climate change and *n* = 3041 for risk evaluation. Political orientation is continuous, gender is dichotomous (men as reference category), and age (age group 15–24 as reference category) and country (UK as reference category) categorical. All reported p-values are based on the Wald test.

*p < .05

**p < .01

***p < .001

## Discussion

The goal of the current paper was to categorize expected climate change impacts according to Böhm and Pfister’s [11] framework for environmental risks. Following the general outline proposed by this framework, individuals may focus on one or more of the five levels when asked in an open-response format what they perceive to be the causes or consequences of climate change (attitudes, motives and goals, actions and activities, emissions and pollution, environmental changes, and/or impacts on humans). The specific aims were to (i) explore the distribution of impact categories across and between four European countries, (ii) predict the mentioning of specific impact categories from socio-demographics, and (iii) investigate the relationship between the impact categories and two types of risk judgments.

### Impact categories

The physical and social environment in a country might generate a shared understanding of both *what* is affected by climate change (e.g., human health vs. environmental damage) and to what degree this is considered a risk [31]. Whether respondents mention changes in actions and activities or impacts on humans as the most important impact might, for example, depend on a countries’ previous climate change experiences and media reporting. This could again influence the cognitive availability and accessibility of examples of climate change consequences to the population [23, 24, 32]. Even though the four countries have different socio-political profiles [27], our results do not support drastic differences between them. Instead, the findings align with literature showing that when laypeople are asked to elaborate on climate change in general, they tend to focus on environmental impacts [33–36]. This pattern was consistent across the four countries investigated in the current study, indicating that certain aspects of people’s causal beliefs about climate change might be somewhat generalizable, independent of socio-political factors [27]. Despite the correspondence regarding the level at which expected climate change consequences were identified, the obtained answers may still differ in content; for instance, we find that people in Germany and France more frequently mention natural disasters.

With regards to the remaining levels, impacts on humans, which would be the most direct consequence of environmental changes according to the framework, was the second biggest category. Not many, even in the key energy-producing countries included in this study, mentioned changes in attitudes, actions, or emissions as the most important impact. However, we found some differences between countries. For example, the category ’action and activities’ turned out to be mentioned more frequently in the UK and Norway compared to Germany and France. Although the differences were not very large, one might speculate that ‘actions and activities’ were mentioned more often in these countries because they can be considered less vulnerable to direct climate change impacts [27].

It should be noted that all four countries included in the current study are western European industrialized countries and that our findings might have limited generalizability outside of this context. Past and anticipated consequences of climate change may be different in other parts of the world, and people might have different associations. In line with this, both the distribution of answers between the six categories and the content of the specific impacts could be different. For example, one study among people from the Western Cape in South Africa found that most interviewees mentioned the area’s ongoing water scarcity and rising food prices as effects of climate change [37], while another study found that air quality was frequently associated with climate change concern among residents in China [3]. Further, since previous research has shown that climate change impacts and impact associations can differ between regions within a country [38], future research could either zoom out to different parts of the world and/or zoom in to different regions within a country.

### Predicting impact beliefs

In addition to differences between countries, we wanted to investigate whether age, gender, and political orientation were related to the consequences people mentioned in the open responses. The results show that, for most of the levels, the socio-demographics were not important predictors. This was apparent both from the (lack of) significance of the coefficients and/or the model fit. One notable exception was category six; men, those belonging to older age groups, and those who place themselves further right on the political spectrum were more likely to answer that climate change will have ‘hardly any’ impacts on their respective countries. This is in line with previous research looking at climate skepticism [e.g., 29, 39]. While mental imagery of future impacts of climate change can be shaped by personal experience with local events such as flooding and changing weather [24], not everyone attributes these events to climate change [32, 40]. One reason for thinking that climate change will have few or no impacts, independently of whether one’s country has been subject to extreme weather events, could be that the mentioned groups are more likely to question the causes and consequences of climate change and less likely to attribute specific weather events to climate change. Such an interpretation is in line with literature highlighting the social and motivated aspects of climate change beliefs [41, 42]. This could mean that increased climate and extreme weather events in the future will have limited influence on climate change acceptance and engagement among these groups unless they change their causal attributions.

### Predicting risk judgments

Differentiating between affective (here: worry) and cognitive (here: risk evaluation) aspects of individual risk judgments are in line with the risk-as-feelings hypothesis that emphasizes that the two can diverge when people are facing uncertain and risky situations [43–45]. Further, while the question about worry referred to judgments of climate change in general, the question about risk evaluation asks specifically about how negative climate change impacts will be on one’s country. While more than 50% of respondents in all four countries answered that the effects of climate change on their country will be ‘more negative than positive’ or ‘entirely negative’, this was far more frequent in France and Germany than in Norway and the UK. Although the two outcomes were positively correlated in general, respondents in Germany reported being less worried about climate change as compared to respondents in France. One reason might be that the question did not specify *when* climate change will have (positive or negative) effects. In line with the concept of psychological distance [5], people’s social, temporal, or hypothetical distance may be high although the question accounts for geographical distance.

With regards to relationships between the six impact categories and the risk judgments, the pattern was relatively similar for both outcomes: impact associations categorized as attitudes and goals (Level 1), environmental changes (Level 4), or impacts on humans (Level 5) were related to both higher levels of worry about climate change and expectations of more negative climate change impacts. Expecting ‘hardly any impacts’ of climate change on one’s country was negatively related to both risk judgments. The positive effects, particularly the effect of Level 4 and Level 5, are in line with previous studies asking about environmental risks [11, 14]. Maybe surprisingly, the largest positive effect was that of attitudes, motives and goals. Due to large differences in the size of each category, we should be careful in drawing conclusions. Still, future studies could investigate this further.

One notable observation was that the two most frequently mentioned categories, ‘environmental changes’ and ‘impacts on humans’, were consistently related to higher levels of worry about climate change as well as evaluating national climate change impacts negatively. Still, the level of worry about climate change was relatively low. Previous research has shown that when asked to consider how climate change will affect their own country, people tend to focus on somewhat remote impacts, such as consequences for plants and animals or future generations [46]. Consequently, there is some reason to assume that the identified associations could have been even stronger if people were more aware of present-day and personally relevant negative consequences of climate change. Future studies could also account for the valence of the responses. Some people might mention country-specific neutral or positive effects, such as opportunities for agriculture. Such responses might weaken the associations with the outcomes, especially regarding the evaluation of how positive or negative country-specific effects will be.

One difference between the two outcomes was answers referring to emissions and pollution (Level 3). Answers falling into this category were related to general climate change worry, but not to country-specific risk evaluation. Another interesting observation is that the category ‘actions and activities’ were not related to any of the risk judgments. This might be because people consider changes in actions and activities as somewhat controllable and therefore less of a risk, an interpretation that is in line with prior studies [15].

The width (how many levels were mentioned) and depth (whether any of the sub-levels were mentioned) of a response were related to worry about climate change, but not to the measure of risk evaluation. While cognitive assessments of a given risk mainly arise from the perceived probability and severity, emotional reactions such as worry are typically more closely related to factors such as the vividness of imagined impacts [45]. Thus, responses including a larger number or more concrete depictions of possible impacts on the national scale might reflect that a respondent has more vivid images of climate change as a risk. Future research could look further into the question of how the vividness of people’s mental images regarding climate change plays a role in shaping individual risk judgments.

Mentioning consequences in a wider range of categories was related to higher levels of worry. The effect of depth was more complex; the first level of depth was positively related to worry, while the second level did not exhibit a statistically significant effect. This is in line with previous studies; while a general and unspecific belief in negative climate change impacts is related to worry about climate change [2], the relationship between knowledge about specific climate change consequences and worry about climate change has been reported to be weak or non-existent [47]. The results can be interpreted to suggest that more general knowledge about climate change impacts can increase risk judgments, whereas very specific knowledge fails to show a similar effect. Further experimental studies are needed to scrutinize the assumption that informing people about the seriousness of a broad range of possible impacts tends to be more promising than focusing on the level of detail.

Sociodemographic variables were included in all models predicting risk judgments. Women and those further left on the political spectrum reported higher levels of worry about climate change and expectations of more negative country-specific climate change impacts.

We only found differences between the youngest and older age groups for risk evaluation, not for general worry; the age group 15–24 expected more negative impacts than the age groups above 45. The models including the levels, and those including depth and width, explained about 14% and 9% of the variance in worry about climate change and about 9% and 8% of the variance in risk evaluation, respectively.

## Conclusions

This study expands upon the existing literature addressing the content and structure of causal beliefs that members of the public keep about climate change. The findings contribute to our understanding of the type of climate change impacts people expect to be important in the country they live in, and how these expectations relate to risk judgments. Open-ended responses were categorized based on a coding scheme reflecting the theoretical model by Böhm and Pfister [11]. This was done based on the assumption that people think about the national impacts of climate change in a variety of different ways, ranging from changes in public attitudes to human suffering. Future research could increase the practical impacts by focusing on alternative approaches, such as categorizing the responses based on proposed communication strategies (e.g. highlighting the negative impacts on health [48] or human culture [49]), or differentiating between ‘correct’ or ‘faulty’ mental models [e.g., 16, 50].

## Supporting information

S1 Table Descriptive statistics and correlations among closed-ended questions included in the analyses (weighted). (DOCX)

S2 Table Coding scheme. (DOCX)

S3 Table Frequencies for each main level and sublevel within the four countries (weighted). (DOCX)

S1 File Coding instructions. (DOCX)

S1 Dataset (DTA)
