# Supplementary material for: Expected climate change consequences and their role in explaining individual risk judgments
Source: PLoS One. 2023 Feb 15;18(2):e0281258. doi: 10.1371/journal.pone.0281258 (PMC9931152; doi:10.1371/journal.pone.0281258)
Supplement: S1 Table — (DOCX) [file pone.0281258.s001.docx]

**S1 Table. Descriptive statistics and correlations among closed-ended questions included in the analyses (weighted).**

|  | *M* | *SD* | Min | Max | 1 | 2 | 3 | 4 | 5 |
| --- | --- | --- | --- | --- | --- | --- | --- | --- | --- |
| 1. Worry about climate change (1 = Not at all worried; 5 = Extremely worried) | 3.00 | 1.07 | 1 | 5 | - |  |  |  |  |
| 2. Risk evaluation (1 = Entirely positive; 5 = Entirely negative) | 3.76 | 0.90 | 1 | 5 | .30*** | - |  |  |  |
| 3. Gender (0 = Male; 1 = Female) |  |  | 0 | 1 | .14*** | .04* | - |  |  |
| 4. Age (in years) | 47.10 | 18.80 | 15 | 93 | .01 | -.08*** | .05** | - |  |
| 5. Political orientation (0 = Left; 10 = Right) | 4.83 | 2.24 | 0 | 10 | -.18*** | -.11*** | -.05** | .09*** | - |

*Note*. Pairwise correlation coefficients.

**p* < .05

***p* < .01

****p* < .001
