# Supplementary material for: Expected climate change consequences and their role in explaining individual risk judgments
Source: PLoS One. 2023 Feb 15;18(2):e0281258. doi: 10.1371/journal.pone.0281258 (PMC9931152; doi:10.1371/journal.pone.0281258)
Supplement: S2 Table — (DOCX) [file pone.0281258.s002.docx]

**S2 Table. Coding scheme.**

| **Code** | | | **Theme** | **Definition** | **Examples** |
| --- | --- | --- | --- | --- | --- |
| **Level 1** | **Level 2** | **Level 3** |  |  |  |
| 1 | | | Attitudes, motives, and goals | The response mentions human attitudes, motives, and goals, which are seen in connection to climate change. | - „Greed is to blame.“ - „We are not thinking of our descendants.“ - „People will finally wake up.“ |
| 2 | | | Actions and activities | The response mentions human actions and activities, which are seen in connection to climate change.  *[This theme includes two subcategories (Level 2), each of which has three further subcategories (Level 3). If actions and activities are mentioned, please inspect each subcategory at Level 2, and consider whether it would be possible to assign the response to one or both of these subcategories. Mark the main theme, “Actions and activities” at Level 1 as well as the relevant subcategories at Level 2 (21 and/or 22) as “1”. Please consider whether it is possible to assign additional themes to the response at Level 3 (211-213 or 221-223), and mark these subcategories as “1” if applicable. If no action/activity is mentioned, you can mark all categories whose code starts with 2 as “0”]* | - „We have to do something.“ |
| 21 | | | Actions and activities related to mitigating climate change | The response mentions human actions and activities that are implemented in order to mitigate climate change. Please also include actions which are seen to be causes of climate change in this category.  *[This theme includes three subcategories (211-213). If actions and activities related to the mitigation of climate change are mentioned, please inspect each subcategory, and consider whether it is possible to assign the response to one or several of these subcategories. Then mark this theme as well as the relevant subcategories as “1”]* | - „We have to prevent climate change.“ |
| 211 | | | Individual actions related to mitigating climate change | The response mentions individual actions that are implemented in order to mitigate climate change. Or: individual actions that are viewed as causes of climate change. | - „People use their car too much.“ - „We will save more energy at home.“ |
| 212 | | | Societal activities related to mitigating climate change | The response mentions societal activities that are implemented in order to mitigate climate change. Or: societal activities that are viewed as causes of climate change. This aspect includes laws, political debates, industrial actions, other economic sectors, etc. | - „Driving bans for cars exceeding critical emission levels“ - „Industrial emissions“ |
| 213 | | | Technological solutions related to mitigating climate change | The response mentions technological solutions that are implemented in order to mitigate climate change. The focus is on the improvement of technologies. | - „Environmentally-friendly energy production, better industrial facilities“ |
| 22 | | | Actions and activities for adapting to climate change | The response mentions actions and activities for adapting to the consequences of climate change.  *[This theme includes three subcategories (221-223). If actions and activities related to the adaption to climate change are mentioned, please inspect each subcategory, and consider whether it is possible to assign the response to one or several of these subcategories. Then mark the theme at Level 2 (22) as well as the relevant subcategories at Level 3 (221-223) as “1”]* | - „We have to adapt.“ |
| 221 | | | Individual actions for adapting to climate change | The response mentions individual actions for adapting to the consequences of climate change. | - „More people will buy air conditioners for coping with hot days.“ |
| 222 | | | Societal activities for adapting to climate change | The response mentions societal activities for adapting to the consequences of climate change. | - „The government will spend more money on coast protection.“ |
| 223 | | | Technological solutions for adapting to climate change | The response mentions technological solutions meant to adapt to the consequences of climate change. The focus is on the development, improvement, or increased use of technologies. | - „We need new techniques for constantly reinforcing dykes.“ |
| 3 | | | Emissions/ pollution | The response mentions emissions and pollution (gases, liquids or other substances that are released into air, water, or soil) related to climate change. | - „CO_2_-emissions and CFCs“ |
| 4 | | | Environmental changes | The response mentions environmental changes. It is not explicitly mentioned whether these changes have negative impact on humans, but it can be implied.  *[This theme includes two subcategories (41-42). If environmental changes are mentioned, please inspect each subcategory, and consider whether it is possible to assign the response to one or both of these subcategories. Then mark the theme at Level 1 as well as the relevant subcategory at Level 2 as “1”]* | - „The earth will warm, polar ice caps will melt.“ - „We hardly have seasons any more.“ - „It will get too hot.“ - „England will turn into a desert.“ |
| 41 | | | Impacts on animals and plants | The response mentions impacts on animals and plants. | - „Crops like corn will benefit, others will yield less harvest. Pests attacking plants will multiply.“ - „We will have tropical mosquitos here.“ |
| 42 | | | Natural disasters | The response mentions possible natural disasters. | - „Tornados, floods and droughts will become common.“ |
| 5 | | | Impacts on humans | The response mentions impacts that affect human beings in particular.  *[This theme includes two subcategories (51-52), each of which comprises further subcategories. If impacts on humans are mentioned, please inspect each subcategory, and consider whether it is possible to assign the response to one or both of these subcategories. Then mark the main theme, “Impacts on humans” at Level 1 as well as the relevant subcategories at Level 2 as “1”. Please then consider whether it is possible to assign additional themes to the response at Level 3, and mark these subcategories as “1” if applicable. If no impacts on humans are mentioned, you can mark all categories whose code starts with 5 as “0”]* | - „There will be severe consequences for us humans.“ |
| 51 | | | Impacts on individuals | The response mentions impacts on single individuals. These consequences do not necessarily have to have an impact on society.  *[This theme includes one subcategory (511 – Impacts on health). If impacts on individuals are mentioned, please consider whether it is possible to assign the response to the subcategory “Impacts on health”. Then mark the theme at Level 2 (51) as well as the relevant subcategory at Level 3 (511) as “1”]* | - „People will die.“ - „Some people will be affected by damage to their houses.“ |
| 511 | | | Impacts on health | The response mentions impacts on the health of individuals. | - „Elderly ladies may suffer circulatory collapse due to extreme heat.“ |
| 52 | | | Impacts on society | The response mentions impacts on society.  *[This theme includes three subcategories (521-523). If impacts on the society are mentioned, please inspect each subcategory, and consider whether it is possible to assign the response to one or several of these subcategories. Then mark the theme at Level 2 as well as the relevant subcategory at Level 3 as “1”]* | - „Tourism could suffer.“ - „Energy supply is threatened.“ |
| 521 | | | Impacts on immigration | The response mentions impacts on immigration and refugee movements. | - „We will face large immigration streams, e.g. from the Netherlands.“ |
| 522 | | | Impacts on the agricultural sector | The response mentions impacts on the agricultural sector. | - „Agriculture will suffer from this.“ |
| 523 | | | Impacts on the economy | The response mentions impacts on the economy. | - „Economic power will decline“ - „Increases in prices for food and consumer goods“ |
| 6 | | | Hardly any impacts | The response indicates that climate change does not (or will not) have any significant impacts on the UK. | - „Nothing will change.“ |
