# Supplementary material for: Expected climate change consequences and their role in explaining individual risk judgments
Source: PLoS One. 2023 Feb 15;18(2):e0281258. doi: 10.1371/journal.pone.0281258 (PMC9931152; doi:10.1371/journal.pone.0281258)
Supplement: S3 Table — (DOCX) [file pone.0281258.s003.docx]

**S4 Table. Frequencies for each main level and sublevel within the four countries (weighted).**

|  | **UK** | | | **Norway** | | | **Germany** | | | **France** | | |
| --- | --- | --- | --- | --- | --- | --- | --- | --- | --- | --- | --- | --- |
|  | Main level | Sublevel 1 | Sublevel 2 | Main level | Sublevel 1 | Sublevel 2 | Main level | Sublevel 1 | Sublevel 2 | Main level | Sublevel 1 | Sublevel 2 |
| **1: Attitudes, motives, and goals** | **14.95 %** |  |  | **0.49 %** |  |  | **1.28 %** |  |  | **1.24 %** |  |  |
| **2: Actions and activities** | **14.80 %** |  |  | **8.66 %** |  |  | **4.72 %** |  |  | **3.66 %** |  |  |
| 21: Actions and activities related to mitigation |  | 12.13 % |  |  | 7.85 % |  |  | 2.55 % |  |  | 2.08 % |  |
| 211: Individual-level mitigation |  |  | 2.26 % |  |  | 2.35 % |  |  | 0.50 % |  |  | 0.82 % |
| 212: Societal-level mitigation |  |  | 5.79 % |  |  | 4.20 % |  |  | 1.59 % |  |  | 0.49 % |
| 213: Technological mitigation |  |  | 3.72 % |  |  | 0.11 % |  |  | 0.95 % |  |  | 0.34 % |
| 22: Actions and activities for adaptation |  | 0.69 % |  |  | 0.54 % |  |  | 1.70 % |  |  | 2.00 % |  |
| 221: Individual-level adaptation |  |  | 0.15 % |  |  | 0.00 % |  |  | 1.30 % |  |  | 0.81 % |
| 222: Societal-level adaptation |  |  | 0.13 % |  |  | 0.18 % |  |  | 0.30 % |  |  | 0.56 % |
| 223: Technological adaptation |  |  | 0.40 % |  |  | 0.00% |  |  | 0.12 % |  |  | 0.11 % |
| **3: Emissions/ pollution** | **12.92 %** |  |  | **6.17 %** |  |  | **2.50 %** |  |  | **7.92 %** |  |  |
| **4: Environmental changes** | **60.94 %** |  |  | **85.93 %** |  |  | **86.81 %** |  |  | **85.92 %** |  |  |
| 41: Impacts on animals and plants |  | 6.12 % |  |  | 9.59 % |  |  | 6.24 % |  |  | 3.57 % |  |
| 42: Natural disasters |  | 9.04 % |  |  | 12.70 % |  |  | 38.41 % |  |  | 40.75 % |  |
| **5: Impacts on humans** | **9.85 %** |  |  | **12.84 %** |  |  | **19.15 %** |  |  | **15.28 %** |  |  |
| 51: Impacts on individuals |  | 4.26 % |  |  | 3.16 % |  |  | 3.11 % |  |  | 6.47 % |  |
| 511: Individual health |  |  | 1.55 % |  |  | 1.02 % |  |  | 1.35 % |  |  | 2.60 % |
| 52: Impacts on society |  | 5.56 % |  |  | 11.07 % |  |  | 13.40 % |  |  | 10.48 % |  |
| 521: Immigration |  |  | 1.25 % |  |  | 2.80 % |  |  | 3.19 % |  |  | 1.99 % |
| 522: The agricultural sector |  |  | 0.61 % |  |  | 4.26 % |  |  | 7.47 % |  |  | 5.47 % |
| 523: The economy |  |  | 2.80 % |  |  | 3.90 % |  |  | 2.78 % |  |  | 1.19 % |
| **Hardly any impacts** | **11.86 %** |  |  | **2.93 %** |  |  | **1.63 %** |  |  | **0.28 %** |  |  |

*Note.* Each answer to the open-ended question could be sorted into several levels. Missing responses were removed before calculating the percentages.
