# Supplementary material for: Expected climate change consequences and their role in explaining individual risk judgments
Source: PLoS One. 2023 Feb 15;18(2):e0281258. doi: 10.1371/journal.pone.0281258 (PMC9931152; doi:10.1371/journal.pone.0281258)
Supplement: S1 File — (DOCX) [file pone.0281258.s004.docx]

**S3 Coding instructions.**

**Coding Instructions**

Dear coder,

Many thanks for your willingness to cooperate on our study!

**Task**

We need help in analysing responses that were given in a survey. The survey dealt with the public perception of climate change among UK citizens. Approximately 1000 people were asked about their personal opinions concerning climate change.

A part of the survey addressed perceived impacts of climate change on the UK. The exact wording of the question in the questionnaire was as follows:

**„Climate change may affect different countries in different ways. What do you think will be the most important effect of climate change on the UK?**

You will deal with the responses to this question. We are interested in finding out what first comes to people’s minds when thinking about the impacts of climate change on the UK.

**The themes studied**

Your task is to analyse the responses with respect to which themes are expressed in them. There are six possible superordinate themes that can be mentioned in a response: (1) Attitudes, motives and goals, (2) actions and activities, (3), emissions, exhaustions or pollutions, (4) environmental changes, (5) impacts on humans, (6) hardly any impacts.

**Material and analysis**

Each response can contain one, several or none of the themes. You will find a detailed description of the various themes in a different Word document („Codingscheme.docx“). Each theme has a code, a definition and one or more examples of typical responses. Please carefully read this list at least twice before starting with the coding so that you get a sense of the different themes. You can refer to this list at any time during coding to orient yourself.

You also have an Excel document („Responses.xlxs“). The first column of this Excel sheet contains the respondents’ answers, one below the other. The columns to the right refer to the various themes; each column refers to one theme and is labelled by the code of the theme. For each response, please mark the themes that are mentioned in the response with 1 and the themes not mentioned with a 0. You will find an example of the coding in the uppermost row of the Excel sheet (row 3, marked in grey).

**Subcategories**

Three of the themes have subcategories that can be used to characterise the responses more specifically. There are two levels of subcategories that differ in how detailed and concrete they are. Together with the superordinate themes this results in three levels. The superordinate themes are on Level 1 and are the most abstract and general. The subcategories on Level 3 are the most detailed or concrete. The three themes that have subcategories are: (2) actions and activities, (4) environmental changes, and (5) impacts on humans). The category structure of these three themes is as follows:

2. Actions and activities

2.1 Actions and activities related to mitigating climate change

2.1.1 Individual actions related to mitigating climate change

2.1.2 Societal activities related to mitigating climate change

2.1.3 Technological solutions related to mitigating climate change

2.2 Actions and activities for adapting to climate change

2.2.1 Individual actions for adapting to climate change

2.2.2 Societal activities for adapting to climate change

2.2.3 Technological solutions for adapting to climate change

4. Environmental changes

4.1 Impacts on animals and plants

4.2 Natural disasters

5. Impacts on humans

5.1 Impacts on individuals

5.1.1 Impacts on health

5.2 Impacts on society

5.2.1 Impacts on immigration

5.2.2 Impacts on the agricultural sector

5.2.3 Impacts on the economy

**Using subcategories**

Each category has its own code, which is listed in the document with the detailed definitions of the themes and categories (Codingscheme.docx). The length of a code (i.e. its number of digits) indicates the level of the category that the code denotes. Superordinate themes have a one-digit code; subcategories on Level 2 have a two-digit code; subcategories on Level 3 have a three-digit-code. In the Excel sheet (Responses.xlsx), each theme and subcategory has a column that is labelled by its code.

If a response mentions the superordinate theme „2. Actions and activities“, mark the column of this theme with a 1. Then read the response again carefully and consider whether the mentioned action/activity serves to mitigate or to adapt to climate change. Mark the subcategory that applies, if any, with a 1. If you have chosen one of these options, consider whether the action/activity is individual, societal or technological. Then mark the column that applies, if any, with a 1. Mark all themes and subcategories that do not apply with a 0. It is possible that one part of a response refers to some action/activity that serves to mitigate climate change and another part of the response refers to an action/activity for adapting to climate change (e.g. “We have to all save energy and to build higher dykes”). Then you would mark both subcategories with a 1. Considering the example above, the coding would be as follows: 2. = 1, 2.1 = 1, 2.1.1 = 1, 2.1.2 = 0, 2.1.3 = 0, 2.2 = 1, 2.2.1 = 0, 2.2.2 = 1, 2.2.3 = 0.

Proceed in the same manner for the superordinate themes “4. Environmental changes” and “5. Impacts on humans”. If an environmental change or some impact on humans is mentioned, look at the subcategories of these themes and mark the codes that apply with a 1. Mark all subcategories that do not apply with a 0.

Please try to code each response on the most concrete level possible (i.e. to assign the lowest possible code). A superordinate theme may entail one, several or none of its subcategories.

Example: A response may be assigned one of the superordinate themes at Level 1 (e.g. theme “5. Impacts on humans”), but none of the subcategories on Level 2 or Level 3. An example would be the response “Humans will have to suffer”. This response expresses that there will be impacts on humans, but it doesn’t specify what these impacts are exactly. In this case, mark only the superordinate theme with 1 and all of its subcategories with 0.

Another example: A response may be assigned a superordinate theme at Level 1 and a subcategory at Level 2 (e.g. the theme “5. Impacts on humans” and the subcategory “”5.1 Impacts on individuals”), but none of the subcategories on Level 3 (e.g. not “5.1.1 Impacts on health”). An example would be the response “Residential houses will be destroyed”. This response expresses an impact that affects individual people but not their health. In this case, mark the superordinate theme at Level 1 and the subcategory at Level 2 with 1 and the subcategory at Level 3 with 0. For the example above the coding would be as follows: 5. = 1, 5.1 = 1, 5.1.1 = 0, 5.2 = 0, 5.2.1 = 0, 5.2.2 = 0, 5.2.3 = 0.

**Final result**

Altogether, there are 22 themes and subcategories. Eventually there should be 0 or a 1 for every response in each of the 22 columns of the Excel spreadsheet.

There is no time limit for your work and you can go back at any time to previous responses that you have already coded if you want to revise your coding. What matters is that your coding is as accurate and thorough as possible. If you have comments, questions or are uncertain about a coding, make a note in the last column “Comment” of the Excel sheet (Responses.xlsx) and give a brief explanation.
